# Systematically higher Ki67 scores on core biopsy samples compared to corresponding resection specimen in breast cancer: a multi-operator and multi-institutional study

**DOI:** 10.1038/s41379-022-01104-9

**Published:** 2022-06-21

**Authors:** Balazs Acs, Samuel C. Y. Leung, Kelley M. Kidwell, Indu Arun, Renaldas Augulis, Sunil S. Badve, Yalai Bai, Anita L. Bane, John M. S. Bartlett, Jane Bayani, Gilbert Bigras, Annika Blank, Henk Buikema, Martin C. Chang, Robin L. Dietz, Andrew Dodson, Susan Fineberg, Cornelia M. Focke, Dongxia Gao, Allen M. Gown, Carolina Gutierrez, Johan Hartman, Zuzana Kos, Anne-Vibeke Lænkholm, Arvydas Laurinavicius, Richard M. Levenson, Rustin Mahboubi-Ardakani, Mauro G. Mastropasqua, Sharon Nofech-Mozes, C. Kent Osborne, Frédérique M. Penault-Llorca, Tammy Piper, Mary Anne Quintayo, Tilman T. Rau, Stefan Reinhard, Stephanie Robertson, Roberto Salgado, Tomoharu Sugie, Bert van der Vegt, Giuseppe Viale, Lila A. Zabaglo, Daniel F. Hayes, Mitch Dowsett, Torsten O. Nielsen, David L. Rimm, on behalf of the International Ki67 in Breast Cancer Working Group of the Breast International Group and North American Breast Cancer Group (BIG-NABCG), Mitch Dowsett, Daniel F. Hayes, Lisa M. McShane, Kelley M. Kidwell, Torsten Nielsen, Samuel Leung, Balazs Acs, Indu Arun, Renaldas Augulis, Sunil S. Badve, Yalai Bai, Anita L. Bane, John M. S. Bartlett, Jane Bayani, Gilbert Bigras, Annika Blank, Signe Borgquist, Henk Buikema, Angela Chan, Martin C. Chang, Carsten Denkert, Robin L. Dietz, Andrew Dodson, Anna Ehinger, Matthew Ellis, Susan Fineberg, Margaret Flowers, Cornelia M. Focke, Chad Galderisi, Dongxia Gao, Abhi Gholap, Allen M. Gown, Carolina Gutierrez, Douglas J. Hartman, Johan Hartman, Judith C. Hugh, Anagha Jadhav, Elizabeth N. Kornaga, Zuzana Kos, Hans Kreipe, Anne-Vibeke Lænkholm, Arvydas Laurinavicius, Richard Levenson, Mauro Mastropasqua, Takuya Moriya, Sharon Nofech-Mozes, C. Kent Osborne, Hongchao Pan, Liron Pantanowitz, Ernesta Paola Neri, Frédérique M. Penault-Llorca, Mei-Yin Polley, Tammy Piper, Mary Anne Quintayo, Tilman T. Rau, David L. Rimm, Stefan Reinhard, Stephanie Robertson, Jason Ruan, Takashi Sakatani, Roberto Salgado, Lois Shepherd, Ian Smith, Joseph Sparano, Melanie Spears, Malini Srinivasan, Jane Starczynski, Tomoharu Sugie, Austin Todd, Bert van der Vegt, Giuseppe Viale, Shakeel Virk, Yihong Wang, Hua Yang, Lila A. Zabaglo, Zhiwei Zhang, Inti Zlobec

**Affiliations:** 1 Department of Pathology, Yale University School of Medicine, New Haven, CT USA; 2 Department of Oncology and Pathology, Karolinska Institutet, Stockholm, Sweden; 3 Department of Clinical Pathology and Cancer Diagnostics, Karolinska University Hospital, Stockholm, Sweden; 4 University of British Columbia, Vancouver, BC Canada; 5 Department of Biostatistics, School of Public Health, University of Michigan, Ann Arbor, MI USA; 6 Tata Medical Center, Kolkata, West Bengal India; 7 Vilnius University Faculty of Medicine and National Center of Pathology, Vilnius University Hospital Santaros Clinics, Vilnius, Lithuania; 8 Department of Pathology and Laboratory Medicine, Emory University School of Medicine, Atlanta, GA USA; 9 Juravinski Hospital and Cancer Centre, McMaster University, Hamilton, ON Canada; 10 Ontario Institute for Cancer Research, Toronto, ON Canada; 11 Edinburgh Cancer Research Centre, Western General Hospital, Edinburgh, United Kingdom; 12 Department of Laboratory Medicine and Pathology, University of Alberta, Edmonton, AB Canada; 13 Institute of Pathology, University of Bern, Bern, Switzerland; 14 Institute of Pathology, Triemli Hospital Zurich, Zurich, Switzerland; 15 University of Groningen, University Medical Center Groningen, Groningen, The Netherlands; 16 Department of Pathology & Laboratory Medicine, University of Vermont Medical Center, Burlington, VT USA; 17 Department of Pathology, Olive View-UCLA Medical Center, Los Angeles, CA USA; 18 UK NEQAS for Immunocytochemistry and In-Situ Hybridisation, London, United Kingdom; 19 Montefiore Medical Center and the Albert Einstein College of Medicine, Bronx, NY USA; 20 Dietrich-Bonhoeffer Medical Center, Neubrandenburg, Mecklenburg-Vorpommern Germany; 21 PhenoPath Laboratories, Seattle, WA USA; 22 Lester and Sue Smith Breast Center and Dan L. Duncan Comprehensive Cancer Center, Baylor College of Medicine, Houston, TX USA; 23 Department of Pathology and Laboratory Medicine, University of British Columbia, Vancouver, BC Canada; 24 Department of Surgical Pathology, Zealand University Hospital, Roskilde, Denmark; 25 Department of Medical Pathology and Laboratory Medicine, University of California Davis Medical Center, Sacramento, CA USA; 26 European Institute of Oncology, Milan, Italy; 27 University of Toronto Sunnybrook Health Sciences Centre, Toronto, ON Canada; 28 Imagerie Moléculaire et Stratégies Théranostiques, UMR1240, Université Clermont Auvergne, INSERM, Clermont-Ferrand, France; 29 Service de Pathologie, Centre Jean PERRIN, Clermont-Ferrand, France; 30 Institute of Pathology, Heinrich Heine University and University Hospital of Duesseldorf, Duesseldorf, Germany; 31 Department of Pathology, GZA-ZNA, Antwerp, Belgium; 32 Peter MacCallum Cancer Centre, University of Melbourne, Melbourne, VIC Australia; 33 Kansai Medical University, Hirakata, Osaka Japan; 34 European Institute of Oncology IRCCS, and University of Milan, Milan, Italy; 35 The Institute of Cancer Research, London, United Kingdom; 36 University of Michigan Rogel Cancer Center, Ann Arbor, MI USA; 37 Academic Department of Biochemistry, Royal Marsden Hospital / Institute of Cancer Research, London, UK; 38 Breast Oncology Program, Department of Internal Medecine, University of Michigan Comprehensive Cancer Center, Ann Arbor, MI USA; 39 National Cancer Institute, Bethesda, MD USA; 40 Department of Biostatistics, School of Public Health, University of Michigan, Ann Arbor, MI USA; 41 Genetic Pathology Evaluation Centre, Department of Pathology and Laboratory Medicine, University of British Columbia, Vancouver, BC Canada; 42 Karolinska Institutet, Stockholm, Sweden; 43 Tata Medical Center, Kolkata, West Bengal India; 44 Vilnius University Hospital Santara Clinics, Vilnius University, Vilnius, Lithuania; 45 Indiana University Simon Cancer Center, Indianapolis, IN USA; 46 Yale University School of Medicine, New Haven, CT USA; 47 Juravinski Hospital and Cancer Centre, McMaster University, Hamilton, ON Canada; 48 Ontario Institute for Cancer Research, Toronto, ON Canada; 49 University of Alberta, Edmonton, AB Canada; 50 Institute of Pathology, Triemli Hospital Zurich, Zurich, Switzerland; 51 Skane University Hospital, Lund University, Lund, Sweden; 52 University Medical Center Groningen, Groningen, The Netherlands; 53 Department of Laboratory Medicine and Pathology, University of Alberta, Edmonton, AB Canada; 54 Department of Pathology & Laboratory Medicine, University of Vermont Medical Center, Burlington, VT USA; 55 Charité Campus Mitte, Berlin, Germany; 56 Department of Pathology, University of Pittsburgh, Pittsburgh, PA USA; 57 UK NEQAS for Immunocytochemistry and In-Situ Hybridisation, London, UK; 58 Baylor College of Medicine, Houston, TX USA; 59 Montefiore Medical Center and the Albert Einstein College of Medicine, Bronx, NY USA; 60 Breast Cancer Research Foundation, New York, NY USA; 61 Dietrich-Bonhoeffer Medical Center, Neubrandenburg, Mecklenburg-Vorpommern Germany; 62 MolecularMD, Portland, OR USA; 63 University of British Columbia, Vancouver, BC Canada; 64 Optra Technologies, NeoPro SEZ, BlueRidge, Hinjewadi, India; 65 Department of Pathology and Laboratory Medicine, University of British Columbia, Vancouver, BC Canada; 66 Lester and Sue Smith Breast Center and Dan L. Duncan Comprehensive Cancer Center, Baylor College of Medicine, Houston, TX USA; 67 University of Pittsburgh, Pittsburgh, PA USA; 68 Department of Oncology-Pathology, Karolinska Institutet, Stockholm, Sweden; 69 Tom Baker Cancer Centre, Alberta Health Services, Calgary, AB Canada; 70 BC Cancer, Vancouver, BC Canada; 71 Medical School Hannover, Institute of Pathology, Hannover, Germany; 72 Zealand University Hospital, Slagelse, Region Sjælland Denmark; 73 University of California Davis Medical Center, Sacramento, CA USA; 74 European Institute of Oncology, Milan, Italy; 75 Kawasaki Medical School, Kurashiki, Okayama Prefecture Japan; 76 University of Toronto Sunnybrook Health Sciences Centre, Toronto, ON Canada; 77 Nuffield Department of Population Health, University of Oxford, Oxford, UK; 78 Department of Pathology & Clinical Labs, University of Michigan, Ann Arbor, MI USA; 79 Translational Laboratories, Tom Baker Cancer Centre, Alberta Health Services, Calgary, AB Canada; 80 Centre Jean Perrin and Université d’Auvergne, Clermont-Ferrand, France; 81 Department of Public Health Sciences, The University of Chicago Biological Sciences, Chicago, IL USA; 82 Edinburgh Cancer Research Centre, Western General Hospital, Edinburgh, United Kingdom; 83 Transformative Pathology, Ontario Institute for Cancer Research, Toronto, ON Canada; 84 Institute of Pathology, Heinrich Heine University and University Hospital of Duesseldorf, Duesseldorf, Germany; 85 Universität Bern Institut für Pathologie, Murtenstrasse, Bern, Switzerland; 86 Nippon Medical School, Bunkyo-ku, Tokyo Japan; 87 Institut Jules Bordet, Brussels, Belgium; 88 Department of Pathology and Molecular Medicine, Queen’s University, Kingston, ON Canada; 89 Ralph Lauren Centre for Breast Cancer Research, The Royal Marsden Hospital, London, United Kingdom; 90 Birmingham Heart of England, National Health Service, Birmingham, United Kingdom; 91 Kansai Medical University, Hirakata, Osaka Japan; 92 University of Milan, Milan, Italy; 93 Queen’s University, Kingston, ON Canada; 94 Rhode Island Hospital, Brown University, Providence, RI USA; 95 The Institute of Cancer Research, London, United Kingdom; 96 Biometric Research Program, Division of Cancer Treatment and Diagnosis, National Cancer Institute, Bethesda, MD USA

**Keywords:** Pathology, Prognostic markers, Breast cancer

## Abstract

Ki67 has potential clinical importance in breast cancer but has yet to see broad acceptance due to inter-laboratory variability. Here we tested an open source and calibrated automated digital image analysis (DIA) platform to: (i) investigate the comparability of Ki67 measurement across corresponding core biopsy and resection specimen cases, and (ii) assess section to section differences in Ki67 scoring. Two sets of 60 previously stained slides containing 30 core-cut biopsy and 30 corresponding resection specimens from 30 estrogen receptor-positive breast cancer patients were sent to 17 participating labs for automated assessment of average Ki67 expression. The blocks were centrally cut and immunohistochemically (IHC) stained for Ki67 (MIB-1 antibody). The QuPath platform was used to evaluate tumoral Ki67 expression. Calibration of the DIA method was performed as in published studies. A guideline for building an automated Ki67 scoring algorithm was sent to participating labs. Very high correlation and no systematic error (*p* = 0.08) was found between consecutive Ki67 IHC sections. Ki67 scores were higher for core biopsy slides compared to paired whole sections from resections (*p* ≤ 0.001; median difference: 5.31%). The systematic discrepancy between core biopsy and corresponding whole sections was likely due to pre-analytical factors (tissue handling, fixation). Therefore, Ki67 IHC should be tested on core biopsy samples to best reflect the biological status of the tumor.

## Introduction

It has been long acknowledged that the immunohistochemical (IHC) detection of Ki67 positive tumor cells provides important clinical information in breast cancer^1^. More recently, Ki67 gained clinical utility in the T1-2, N0-1, estrogen receptor-positive (ER) and HER2-negative patient group by allowing to identify those patients that are unlikely to benefit from adjuvant chemotherapy^2^. However, Ki67 has not been consistently adopted for clinical care due to unacceptable reproducibility across laboratories^3–5^.

Therefore, the International Ki67 in Breast Cancer Working Group (IKWG) originally published consensus recommendations in 2011 for best practices in the application of Ki67 IHC in breast cancer^6^. According to this consensus, parameters that predominantly influence Ki67 IHC results can be grouped into pre-analytical (type of biopsy, tissue handling), analytical (IHC protocol), interpretation and scoring, and data analysis steps^6^. As the scoring method was the largest contributor to test variability^7^, the IKWG has undertaken serious efforts to standardize the Ki67 scoring method of pathologists^8,9^. Although in multi-institutional studies, standardized Ki67 scoring methods reached pre-defined thresholds for adequate reproducibility^9,10^, this was only after completing calibration training and by using tedious counting methods. In this context, recently updated guidelines by the IKWG now recommend Ki67 IHC for clinical adoption in specific situations, including the identification of very low (<5) or very high proliferation (>30) indices, that render more expensive gene expression tests unnecessary^2^.

An important additional issue that can cause variability in Ki67 measurements is the type of specimen (core biopsy vs excision) and its effect on Ki67 scoring in a multi-center setting^2^. Indeed, the IKWG recommended use of core biopsies (CB), based on apparent superior results for Ki67 when visual evaluation was compared to that of whole sections (WS).

In this multi-observer and multi-institutional study, we aimed to investigate the comparability of Ki67 measurements across corresponding core biopsy and resection specimens from the same breast cancer cases, when evaluated using a calibrated, automated reading system. Furthermore, we assessed between-(consecutive) section differences in Ki67 scoring as no difference between sections will facilitate the selection of the tumor-block to perform the IHC staining on.

## Materials and methods

### Patients

Thirty cases of ER-positive breast cancer used in phase 3 of IKWG initiatives collecting 15 cases from the UK and 15 cases from Japan designed to cover a range of Ki67 scores^9^ were employed in this study. No outcome data were collected for this cohort. Patient selection was irrespective of patients’ age at diagnosis, grade, tumor size or lymph node status. The clinicopathological characteristics of these 30 cases can be found in our previous publications^9,10^.

### Tissue preparation and immunohistochemistry (IHC)

Tissues from UK patients, both core biopsies and surgical resections were collected according to ASCO/CAP guidelines, while patients’ tissues from Japan were collected following ISO (International Organization for Standardization) 15189 approved by the Japan Accreditation Board. Preparation of the Ki67 slides of the first cohort has been previously described^9^. Briefly, the corresponding core-cut biopsy and surgical resection blocks were centrally cut and stained with Ki67, resulting in 60 Ki67 slides from 30 cases. The IHC was performed using monoclonal antibody MIB-1 at dilution 1:50 (DAKO UK, Cambridgeshire, UK) using an automated staining system (Ventana Medical Systems, Tucson, AZ, USA) according to the consensus criteria established by the International Ki67 Working Group^6^. Sections from the same block were stained in a single immunohistochemistry run, except for four cases where the staining was performed in two different runs. This approach effectively controls for any technical variation in staining.

### Sample distribution

Twenty volunteer pathologists from 15 countries, most of whom participated in the previous Phase 3A study, were invited to participate. Four adjacent sections from each of the 60 blocks were centrally stained as follows: the first section with haematoxylin and eosin (H&E), the second with p63 (a myoepithelial marker, to assist the distinction of DCIS from invasive breast cancer) and the third to fourth with Ki67 (designated as slide sets 1–2).

**Fig. 1:**
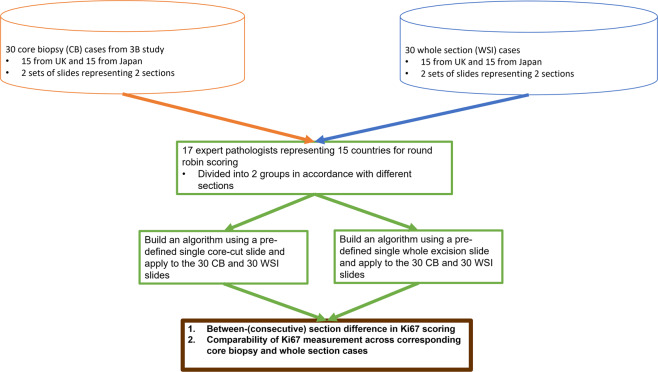
Study design. Thirty patients of ER-positive breast cancer were enrolled comprising 15 cases from UK and 15 cases from Japan. Corresponding core-cut biopsy and surgical resection blocks were centrally cut two adjacent sections per case and stained with Ki67. Seventeen pathologists from 15 countries were given 60 slides (30 Core cut biopsy slides and 30 surgical resection specimen slides) of Ki67 to score.

The Aperio ScanScope XT platform was used at 20× magnification to digitize the slides (pixel size: 0.4987 µm × 0.4987 µm), which were uploaded to a server and distributed as digital images. Seventeen pathologists successfully completed the study (Fig. 1).

### Digital image analysis (DIA)

**Fig. 2:**
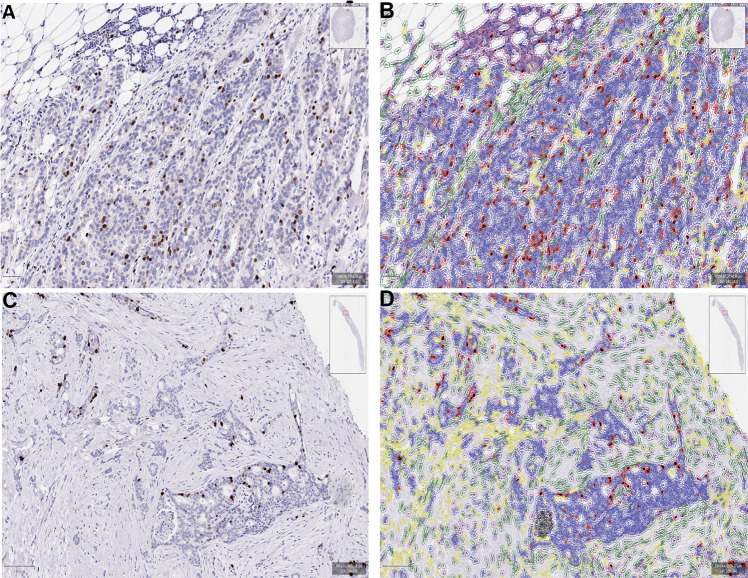
Digital Image Analysis. Representative pictures of digital image analysis (DIA) masks on a resection specimen (**A**, **B**) and a core biopsy case (**C**, **D**). Blue corresponds to Ki67 negative tumor cells, red indicates Ki67 positive tumor cells, green indicates stromal cells and purple marks immune cells. Black corresponds to necrosis and yellow marks other detections (false cell detections, noise).

The QuPath open-source software platform was used to build automated Ki67 scoring algorithms for breast cancer^11^. A detailed guideline for setting up and building an automated Ki67 scoring algorithm was sent to the participating labs. All the participating labs were requested to build their own Ki67 scoring algorithm following the instructions and apply them on these 60 slides. The complete step by step instructions are available in Supplementary File 1. The reason why we asked each lab to build their own algorithm instead of using the same pre-trained and locked down Ki67 algorithm was to mimic clinical practice. As of the date of the study, no generalizable Ki67 scoring algorithm was available that provides whole slide scoring. Thus, theoretically, all the labs would need to adjust/ optimize any such DIA approach to their lab characteristics (different fixation, different antibodies and IHC protocols etc.) necessitating a lab-specific DIA approach. Calibration of the DIA method/guideline was performed in our previous studies demonstrating very good reproducibility among users^12,13^. Briefly, after the whole invasive cancer area on a digitized slide was annotated, hematoxylin and DAB stain estimates for each case were refined using the “estimate stain vectors” command. We used watershed cell detection^14^ to segment the cells in the image with the following settings: Detection image: Optical density sum; requested pixel size: 0.5 µm; background radius: 8 µm; median filter radius: 0 µm; sigma: 1.5 µm; minimum cell area: 10 µm^2^; maximum cell area: 400 µm^2^; threshold: 0.1; maximum background intensity: 2. In order to classify detected cells into tumor cells, immune cells, stromal cells, necrosis and others (false detections, background) (Supplementary File 1), we used random trees as a supervised machine-learning method. The features used in the classification are described in Supplementary Table 1. After setting the optimal color deconvolution and cell segmentation, two independent classifiers were trained on a randomly selected, pre-defined core biopsy (CB classifier) and a resection specimen slide (WSI classifier). Both CB and WSI classifiers were run on both CB slides and resection specimen slides in order to adjust for potentially different characteristics of the two specimen types (Fig. 2).

### Statistical analysis

For statistical analysis, SPSS 22 software (IBM, Armonk, USA) software was used. Degree of agreement was evaluated by Bland–Altman plot and linear regression. To assess differences between specimen type the Wilcoxon signed-rank test was applied, since the data were not normally distributed. Data were visualized using boxplot, spaghetti plot, and dot-plot.

## Results

### Between-(consecutive) section difference in Ki67 scoring

**Fig. 3:**
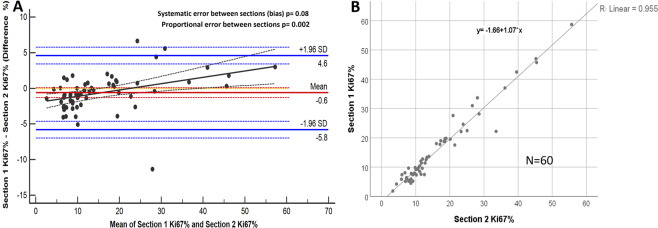
Between-(consecutive) section difference in Ki67 scoring. Bland–Altman plot comparing Ki67 scores between consecutive sections (**A**). Orange dashed line corresponds to the expected mean zero difference between Ki67 scores of the two sections. Red line represents the observed mean difference between Ki67 scores of the two sections, namely the observed bias (red dashed lines are the CI of the observed mean difference). Blue lines illustrate the range of agreement (lower and upper limit of agreement) based on 95% of differences (blue dashed lines are the CI of the limits of agreement). Black line is the fitted regression line to detect potential proportional error (black dashed lines are the CI of the regression line). **B** represents the scatter plot with fitted regression between the Ki67 scores of the two consecutive sections.

Very high correlation and no systematic error (bias: −0.6%; *p* = 0.08) was found between the two consecutive (serial) sections regarding Ki67 scores. If the Ki67 score is higher for a given case, the difference between the sections tends to be also greater (proportional error *p* = 0.002, Fig. 3.), however this difference (0.6% mean difference) does not reach clinical relevance.

### Specimen type (CB vs resection specimen) difference in Ki67 scoring

**Fig. 4:**
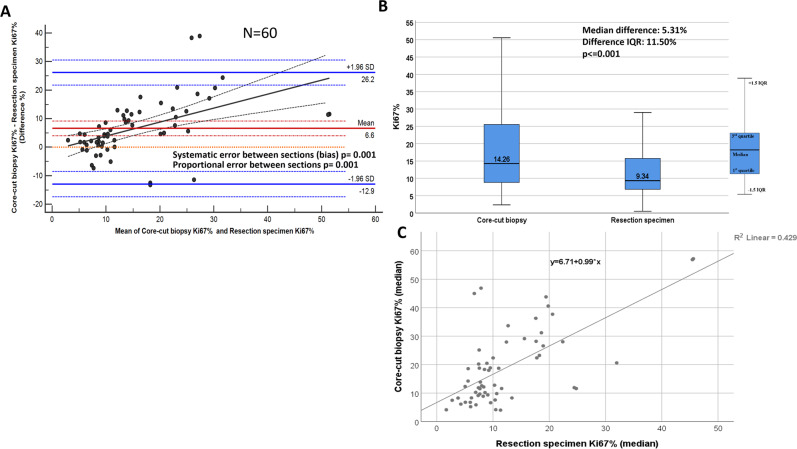
Between-specimen (CB vs resection specimen) difference in Ki67 scoring. Bland–Altman plot comparing Ki67 scores between specimens (**A**). Orange dashed line corresponds the expected mean zero difference between Ki67 scores of the two sections. Red line represents the observed mean difference between Ki67 scores of the two sections, namely the observed bias (red dashed lines are the CI of the observed mean difference). Blue lines illustrate the range of agreement (lower and upper limit of agreement) based on 95% of differences (blue dashed lines are the CI of the limits of agreement). Black line is the fitted regression line to detect potential proportional error (black dashed lines are the CI of the regression line). **B** shows the distributions of Ki67 scores of the two specimens. The bottom/top of the boxes represent the first (Q1)/third (Q3) quartiles, the bold line inside the box represents the median and the two bars outside the box represent the lowest/highest datum still within 1.5× the interquartile range (Q3–Q1). **C** represents the scatter plot with fitted regression between the Ki67 scores of the two specimens.

**Fig. 5:**
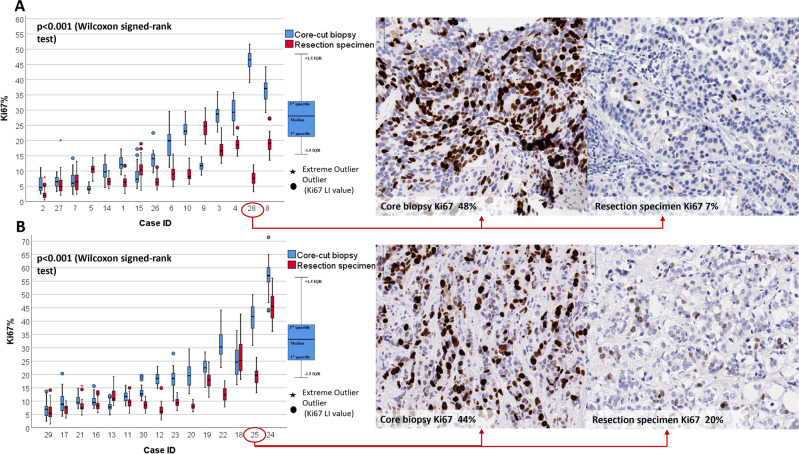
Between-specimen (CB vs resection specimen) difference in Ki67 scoring by case and by origin of the cases. **A** represents cases collected in the United Kingdom with representative Ki67 IHC images of corresponding CB and resection specimens. **B** represents cases collected in Japan with representative Ki67 IHC images of corresponding CB and resection specimens. The bottom/top of the boxes represent the first (Q1)/third (Q3) quartiles, the bold line inside the box represents the median and the two bars outside the box represent the lowest/highest datum still within 1.5× the interquartile range (Q3–Q1). Outliers are represented with circles, extreme outliers with asterisk.

A low correlation was found between core biopsy and whole section excision images (Fig. 4). Ki67 scores were higher when determined on core biopsy slides compared to paired whole sections (*p* ≤ 0.001; median difference: 5.31%; IQR: 11.50%) from subsequent surgical excisions of the same tumor. Systematic error occurred between specimens from the same patient as core biopsy Ki67 scores were greater, with a clinically relevant mean difference of 6.6% (bias *p* = 0.001). The limits of agreement also have to be considered wide from a clinical perspective (between −13.7 and 27). Furthermore, Ki67 scores on CB were even higher compared to WS on cases with higher Ki67 scores (proportional error *p* = 0.001). Moreover, the variability of differences in Ki67 scores between CB and WS showed an increasing trend, proportional to the magnitude of Ki67 score (Fig. 4). The same results were found irrespective of the origin of the specimens (CB vs WS *p* < 0.001 for both UK and Japan cases Fig. 5).

## Discussion

In this study, we observed that clinically relevant and systematic discrepancies occurred in Ki67 scores between core biopsy and corresponding surgical specimens when evaluated with an automated reading system. Overall, Ki67 scores were higher on CB compared to WS samples. Furthermore, this discrepancy was even more pronounced in tumors that expressed higher levels of Ki67 in general.

Ki67 is one of the most promising yet controversial biomarkers in breast cancer with limited adoption into clinical practice due to its high inter- and intra-laboratory variability^3,15^. However, Ki67 is widely used in many countries, there is wide variability in its use (to distinguish luminal A-like vs B-like tumors; to determine whether to decide for gene-expression profiling or not; as an adjunct to mitotic counts, etc.), with still no uniformity between clinicians on how to use this biomarker, let alone which cut-off to use. Although the IKWG set up a guideline in 2011 to improve pre-analytical and analytical performance, inter-laboratory protocols still demonstrated low reproducibility related to different sampling, fixation, antigen retrieval, staining and scoring methods^6,7^. As the latter was the largest single contributor to assay variability, the IKWG has undertaken multi-institution efforts that have standardized visual scoring of Ki67 in a manner which requires on-line calibration tools and careful scoring of several hundred cells, which may or may not be ideal for pathologists in daily practice with time-constraints^8,9^. This result suggests that digital solutions may still be required to address this issue.

The rise of digital image analysis (DIA) platforms has improved capacity and automation in biomarker evaluation^16,17^. DIA platforms are able to assess nuclear IHC biomarkers such as Ki67, and numerous studies have been conducted to compare human visual scoring with DIA platforms^12,18–28^. Although the latest guideline of IKWG recommends Ki67 for clinical practice in specific situations, the type of specimen as a potential pre-analytical factor contributing to Ki67 variability was not specifically investigated in a multi-operator/multi-center setting. In this study we aimed to address these biospecimen questions including assessment by specimen type and between serial sections.

One explanation for our finding would be the presence of tumor heterogeneity, and the broader field of review in a whole section from resection specimen. However, one would expect that this cause of discrepancy would result in random discordance, not the consistent finding that Ki67 scores on core biopsies are higher than that of on resection specimens. Rather, we conclude that lower Ki67 in resection specimens is more easily explained by pre-analytical factors. For example, since longer times to fixation occur with resection specimens compared to CB, persistent cell division will occur even in an unfixed, hypoxic environment. Further, epitope degradation also occurs with prolonged time to fixation^29–31^.

In addition, one can expect that hot spot scoring might lead to less discrepancy between CB and WS because it considers only the hottest area of Ki67 positivity (highest percentiles of Ki67 distribution) on both specimen types, while global assessment evaluates the total Ki67 distribution which can be variable^10^. However, there remains a fundamental issue of exact hot spot definition and where pathologists set its boundaries. Moreover, the International Ki67 Working Group has recommended global scoring over hot spot as it did show a consistent trend towards increased reproducibility in both core biopsy^9^ and excision^10^ specimens.

Additional support for the conclusion that the difference in Ki67 between CB and WS is provided by the observation of clinically relevant differences between specimens in cases from different institutions used in this study, independently scored multiple times by 17 pathologists. Although many studies focused on assessing the level of agreement between CB and resection samples in Ki67 scoring; consensus was not possible due to lack of standardization^32^.

Our results are consistent with previous results showing poor/moderate concordance (κ = 0.195–0.814) occurring between CB and resection specimen in Ki67 scoring^1,33–46^. However, some studies showed higher Ki67 scores on resection samples^35,36,38^. This discrepancy among studies may be due to lack of standardization in methodology leading to different scoring methods, which we have previously demonstrated to be highly variable^2^. Moreover, inter-institutional discrepancies could also be the result of different antibodies and protocols used to detect Ki67, different tissue handling/fixation protocols and at some point tumor Ki67 heterogeneity since Ki67 is heterogeneous in tumors^6^. Thus, our findings provide further support to the latest IKWG recommendations and provide a consensus that Ki67 should be ideally tested on CB samples because it minimizes many fixation problems as Ki67 IHC is more sensitive than ER or HER2 to variabilities in fixation^2^. Since pre-analytical factors are critical in diagnostic pathology, the IKWG recommends that breast cancer samples for Ki67 testing should be processed in line with ASCO/CAP guidelines^2^.

There are a number of limitations in this study. This study only focused on analytical and preanalytical questions, therefore we cannot demonstrate the clinical validation of the calibrated tool. There are many other studies that address the prognostic or predictive value of this test, and that goal was beyond the scope of this effort. For the same reason, further clinical studies are needed to demonstrate how does this consistent difference in Ki67 between corresponding core-cuts and resection specimen impact on prognostic value or its clinical implication on the assessment of neoadjuvant endocrine therapy benefit. Furthermore, the low correlation suggests a critical difference between a core biopsy score and a whole section excision score, which can undermine the use of data on outcome, derived predominantly from resection samples, to identify patients at high risk using a score derived from a core biopsy. Therefore, this study suggests caution in this approach given that even without intervening therapy a clinically relevant change in Ki67 may occur. Further, the Ki67 assessments were based on biospecimens from only 2 central sites. While the participating pathologists within the IKWG represented 15 countries, specimens were centrally acquired and stained. Whereas other investigators have compared specimens from multiple different sites^5,7,47^ we limited the number of sites to remove the variables associated with the technical aspect of the stain. Finally, while the core cut biopsy and resection are from the same case, only a single core was assessed. Thus, we could be missing heterogeneity seen in larger resection specimens. The effect of heterogeneity could be decreased by taking multiple core cuts when clinical situation allows. However, since examination of a single core cut represents the clinical standard of care in several countries, we did not pursue multiple cores.

In conclusion, while we find no significant difference in digitally-assessed Ki67 index between serial sections, we do find a systematic discrepancy between core biopsy and corresponding whole sections – core biopsy samples yield higher scores (likely due to pre-analytical factors including more standard and prompt tissue handling, fixation, etc.). Therefore, this work suggests that Ki67 IHC tested on core biopsy samples should be preferred to excision specimens in clinical decision-making, because doing so will preclude many pre-analytical factors.

## Supplementary information

Supplementary File 1

Supplementary Table 1

## Data Availability

The datasets used and/or analyzed during the current study are available from the corresponding author on reasonable request.
