# Supplementary material for: Systematically higher Ki67 scores on core biopsy samples compared to corresponding resection specimen in breast cancer: a multi-operator and multi-institutional study
Source: Mod Pathol. 2022 Jun 21;35(10):1362–9. doi: 10.1038/s41379-022-01104-9 (PMC9514990; doi:10.1038/s41379-022-01104-9)
Supplement: Supplementary file 1 [file 41379_2022_1104_MOESM1_ESM.pdf]

Supplementary File 1

QuPath protocol for Ki67 IHC evaluation on core biopsy slides and on whole slides

Balazs Acs and David L. Rimm

December 14th, 2018

Version 2.0

International Ki67 in Breast Cancer Working Group

Task: Set up and Use of QuPath for Ki67 evaluation on 30 core biopsy -and 30 whole section slides stained with Ki67 and visualized with DAB on Ventana platform in Breast Cancer.

Download QuPath.

Supported image formats:

- Whole slide image formats:
  - Aperio (.svs, .tif)
  - Hamamatsu (.vms, .vmu, .ndpi)
  - Leica (.scn)
  - MIRAX (.mrxs)
  - Philips (.tiff)
  - Sakura (.svslide)
  - Trestle (.tif)
  - Ventana (.bif, .tif)
  - Generic tiled TIFF (.tif)
  - Perkin Elmer (.qptiff)
- ImageJ TIFF
- JPEG
- PNG

Recommended specification:

- Multi-core 64-bit processor & operating system (e.g. Intel i7)
- 12 GB RAM
- Discrete graphics card
- Solid state hard disk
- Lots of hard disk space to store images and data - may require more than 1 GB to store data related to a single slide

To create a project, choose *File/ Project../ Create new project.* You will then have to select an empty folder in which the project will be made. You should create 2 separate projects: One for the whole slide cases and one for the core biopsy cases.

To add the 30 slides to the current project, click *File/Project../Add images*, then use *Choose files* to select the 30 slides, next click *Import.*

You can create an *images* folder inside your project folder, and copy (or move) your images inside it. In this case, everything will be kept in the same folder - and continue to work, no matter where you move the project folder.

The next time you want to open your project, choose *File/ Project../ Open project,* then go to your project folder and select the *project.qpproj* file.

Supplementary File 1

Step 1: Annotation objects

Start with the slide 56_MIB1 for core biopsy cases and slide 4_MIB1 for whole section cases.

Annotate the whole tissue on each slide with the 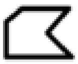 polygon tool, the 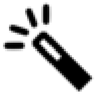 wand tool or the 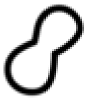 brush tool (left click + dragging the mouse across the image.)

To delete an annotation, go to *Annotations* tab, select the annotation with left click then left click on delete. Try to annotate the entire relevant tissue as close as you can. Avoid damaged tissue at the edges of the specimens. If you see areas of normal tissue, DCIS or artifacts without any considerable area of invasive tumor, you should avoid them when making the large annotation. If you see larger number of normal epithelium, DCIS and artifacts (e.g.: irrelevant DAB staining) within invasive tumor areas, please refer to Step 4, line 100-104.

Step 2: Estimate stain vectors

It is recommended to refine the stain estimates for each new image.

You should first find a representative region containing clear examples of the stains that you want - along with an area of background. Draw an annotation with 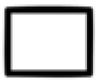 rectangle tool around a region containing examples of each stain and background (whitespace). You should try to choose a small region because in case of a large region QuPath will have to downsample it (by averaging adjacent pixels - which dilutes the useful information) to look for the stains.

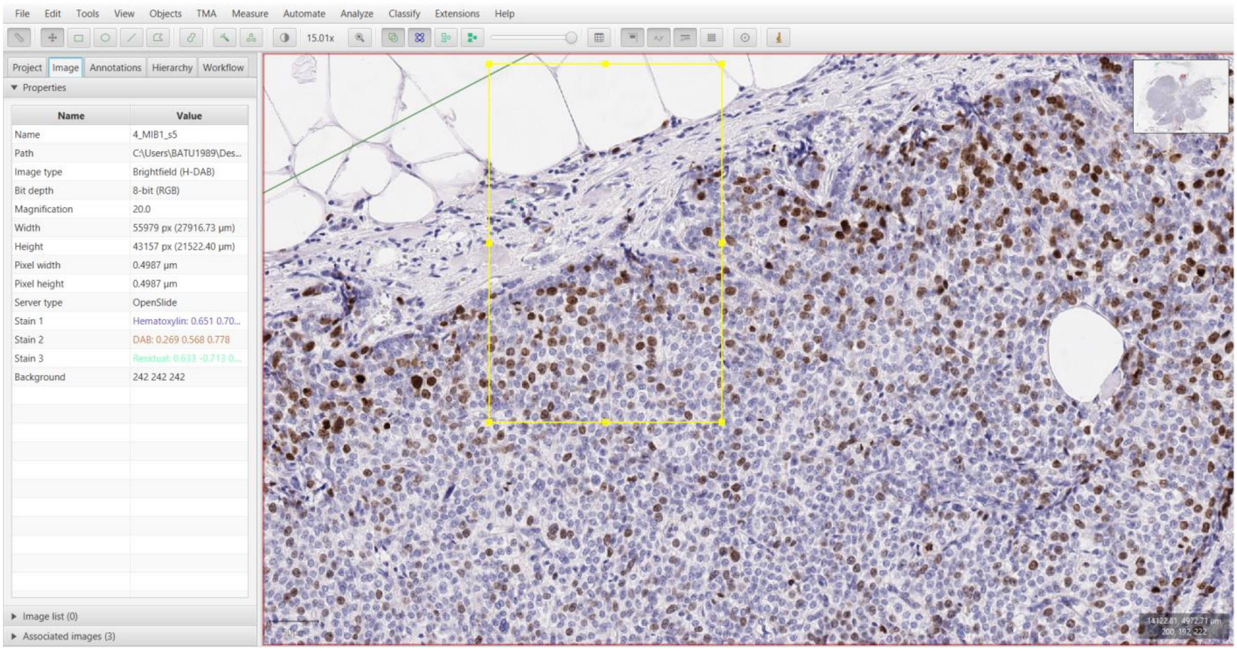

You should try to capture all staining intensities. If this is not possible in a small rectangle, choose a high staining intensity region.

Then, go to *Analyze/Preprocessing/Estimate stain vectors*

If the background values in the drawn region do not match with the background values currently being used, QuPath will ask you whether you want to update the stored values. Assuming that the region you have drawn does contain a representative area of background, you should click *Yes*.

Click on *Auto*, check the updated stain vectors then *OK*. Then, set a name for the updated stain vectors.

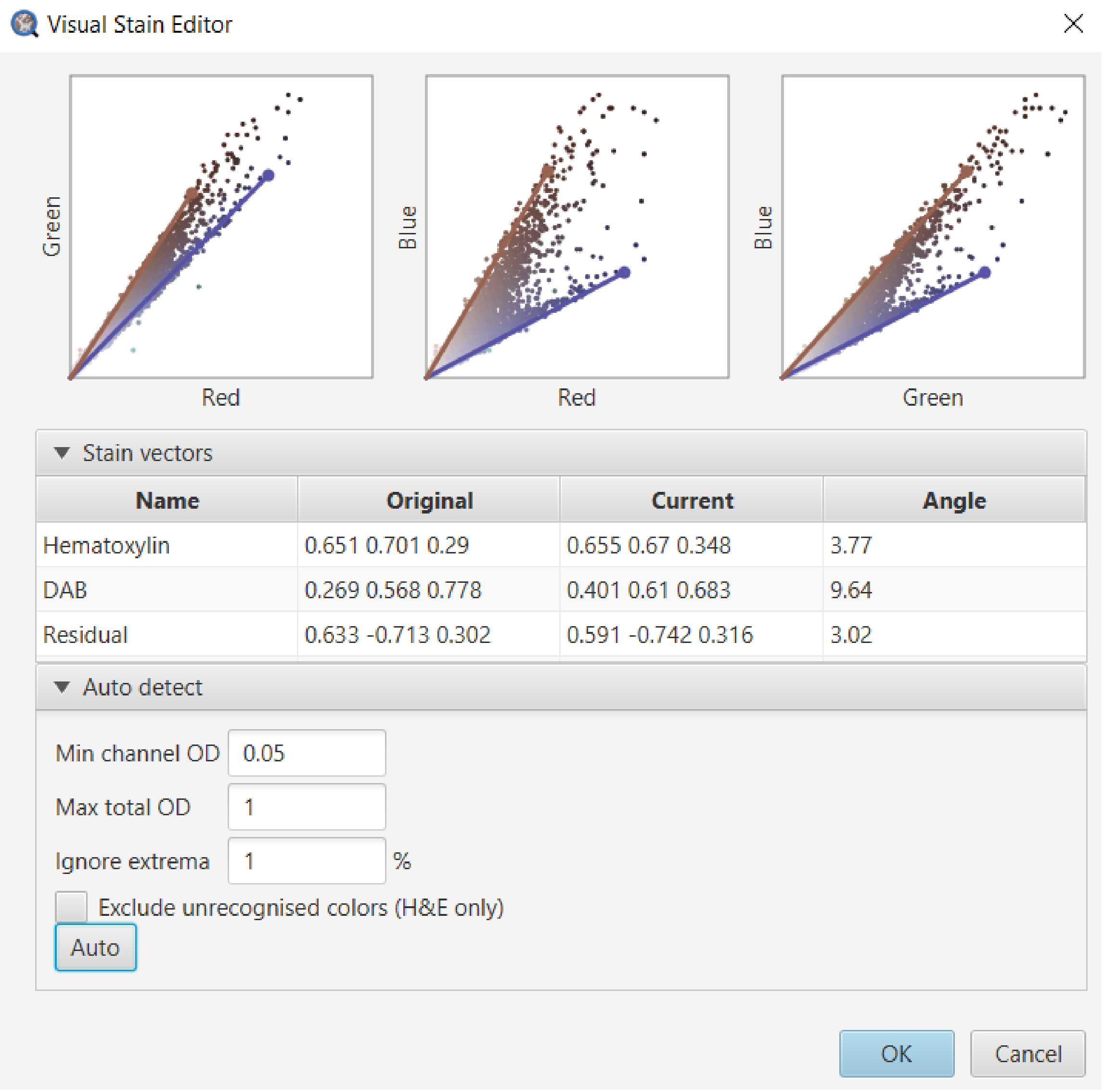

Step 3: Cell detection

You should select the original, large annotation containing the whole tissue, then go to *Analyze/Cell analysis /Cell detection.* This will bring up a dialog, where most of the options relate to how the cells are detected. Choose *Optical density sum* (at *"Choose detection image"*), other default values are good for Ki67 IHC in breast cancer. Click *Run*.

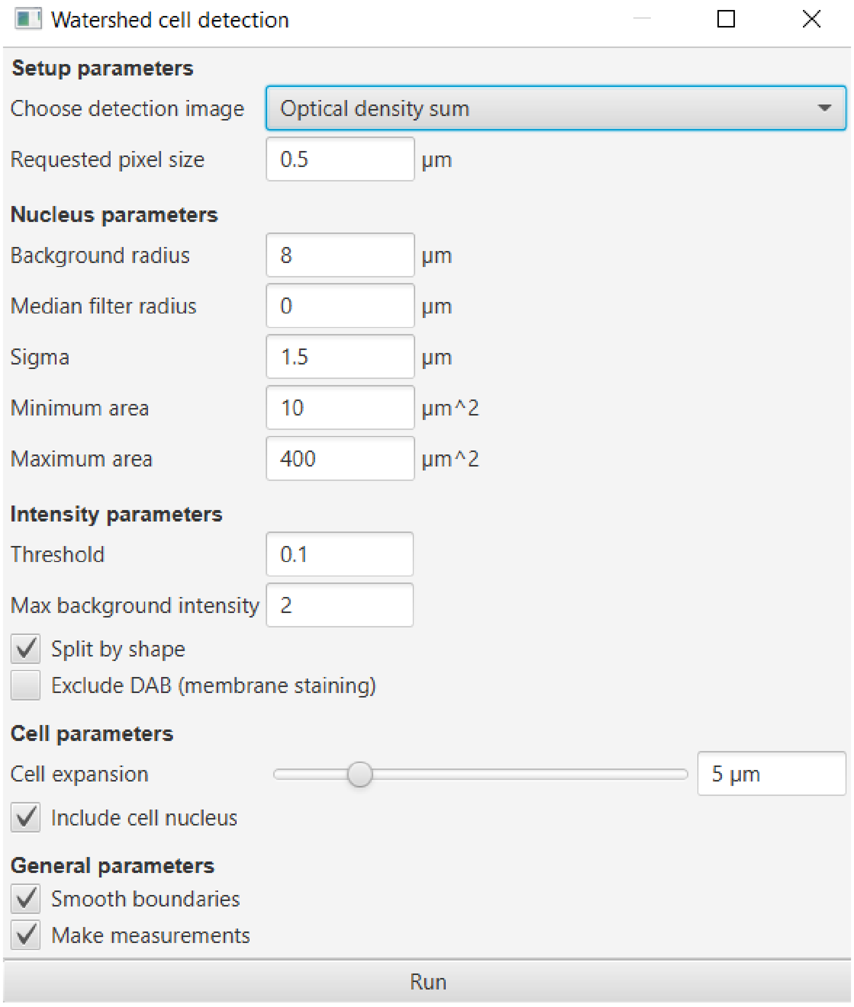

The use of optical density will result the detection of stronger background, blood cells. To handle this, refer to Step 4, line 97-98.

To help QuPath perform an accurate classification it is useful to supplement the existing measurements of individual cells with some additional features that take into consideration more contextual information. Thus, run the *Analyze/ Calculate features/Add smoothed feature* command at 25 µm and 50µm.

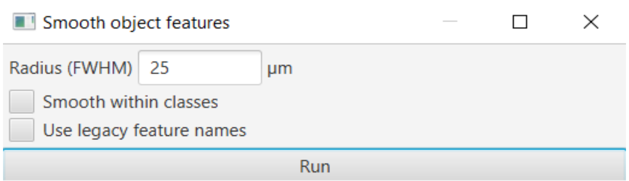

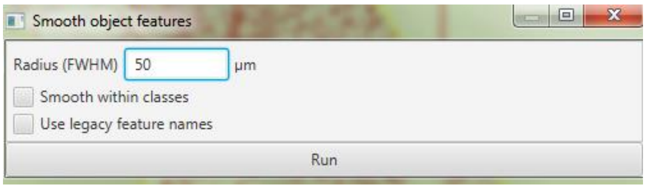

Step 4: Create classification

You should build 2 separate classifiers: One for core biopsy slides and one for whole slides

To set up a classifier for core biopsy (CB) slides use the slide 56_MIB1.

To set up a classifier for whole slides (WSI) use the slide 4_MIB1.

The next step is to begin annotating regions according to classes as follows: Tumor cells, Stroma cells, Immune cells, Necrosis, Other. To annotate regions for classification, use the 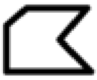 polygon tool. It does not matter whether the detected cells are shown or hidden on the image at the time; however, it can be helpful to toggle the detections on and off with the 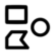 Show/hide detection objects tool while annotating. Try to annotate regions where the cells are detected correctly (shape, size, cell count etc.). Use smaller annotations across the whole slide to avoid the inclusion of different cell types and to represent more areas of the slide. Try to annotate as many regions to represent the whole pattern of the slide and do not over- or underrepresent any areas of the slide.

After an annotation has been drawn, right-click within the drawn annotation, then *Set class* and assign to the appropriate class (Tumor, Stroma, Immune cells, Necrosis and Other).

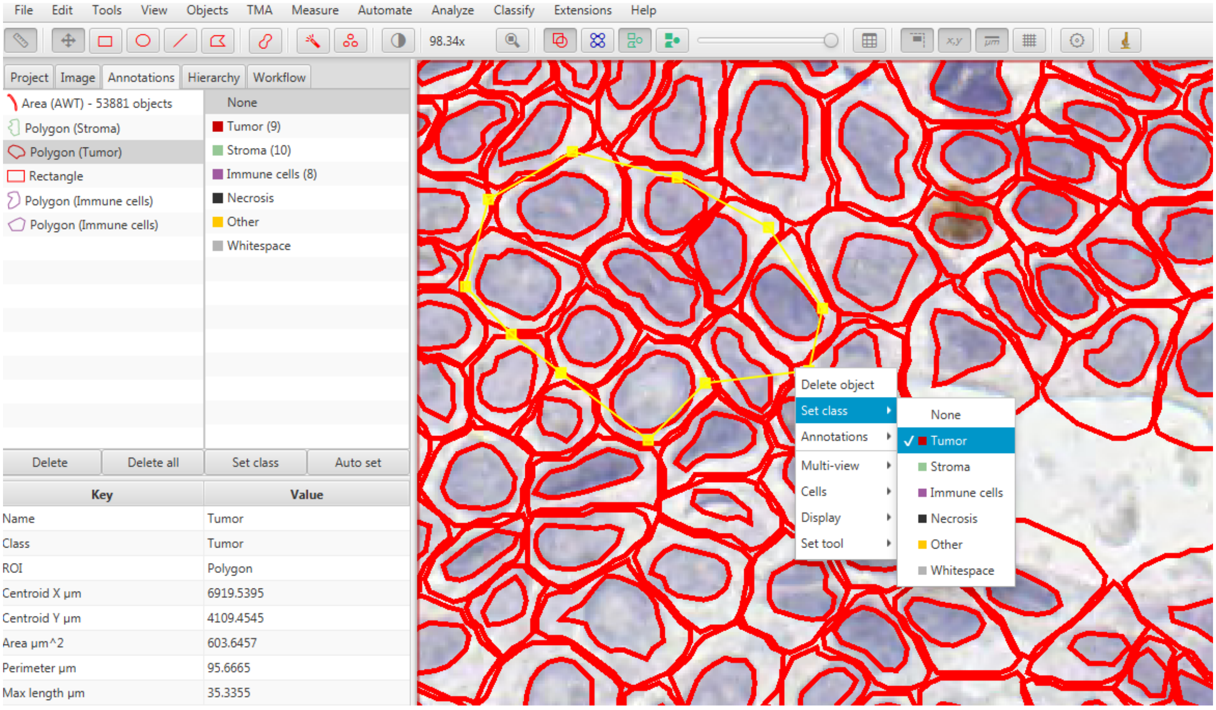

You should see the number increase beside the class that you selected (top right). This is the number of cells inside all the annotations that you have drawn and assigned to this class.

Supplementary File 1

Annotate examples of necrosis (see images below) and assign them to *Necrosis* class.

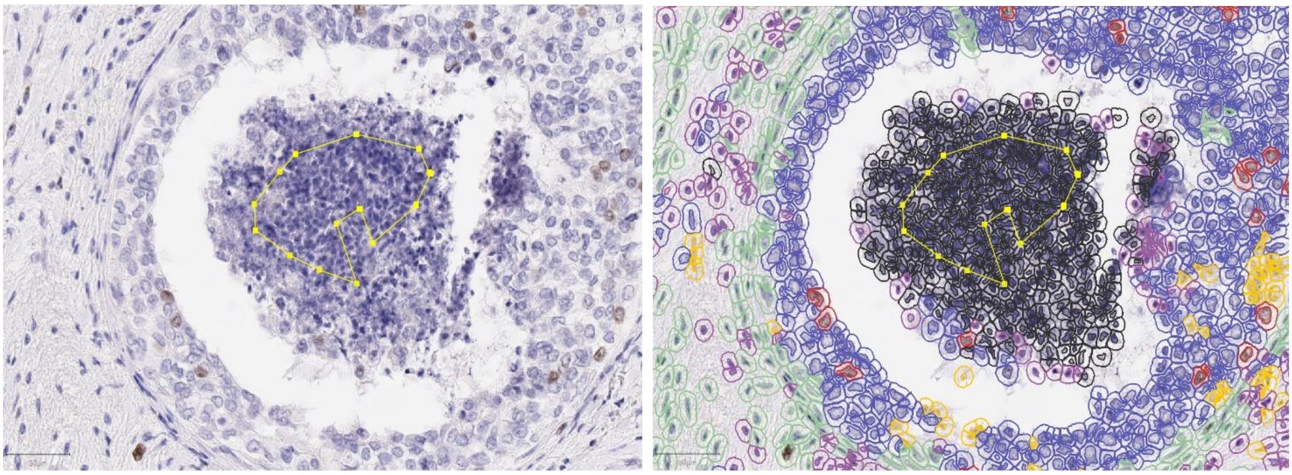

Annotate examples of false cell detections (see images below) and a sign them to *Other* class.

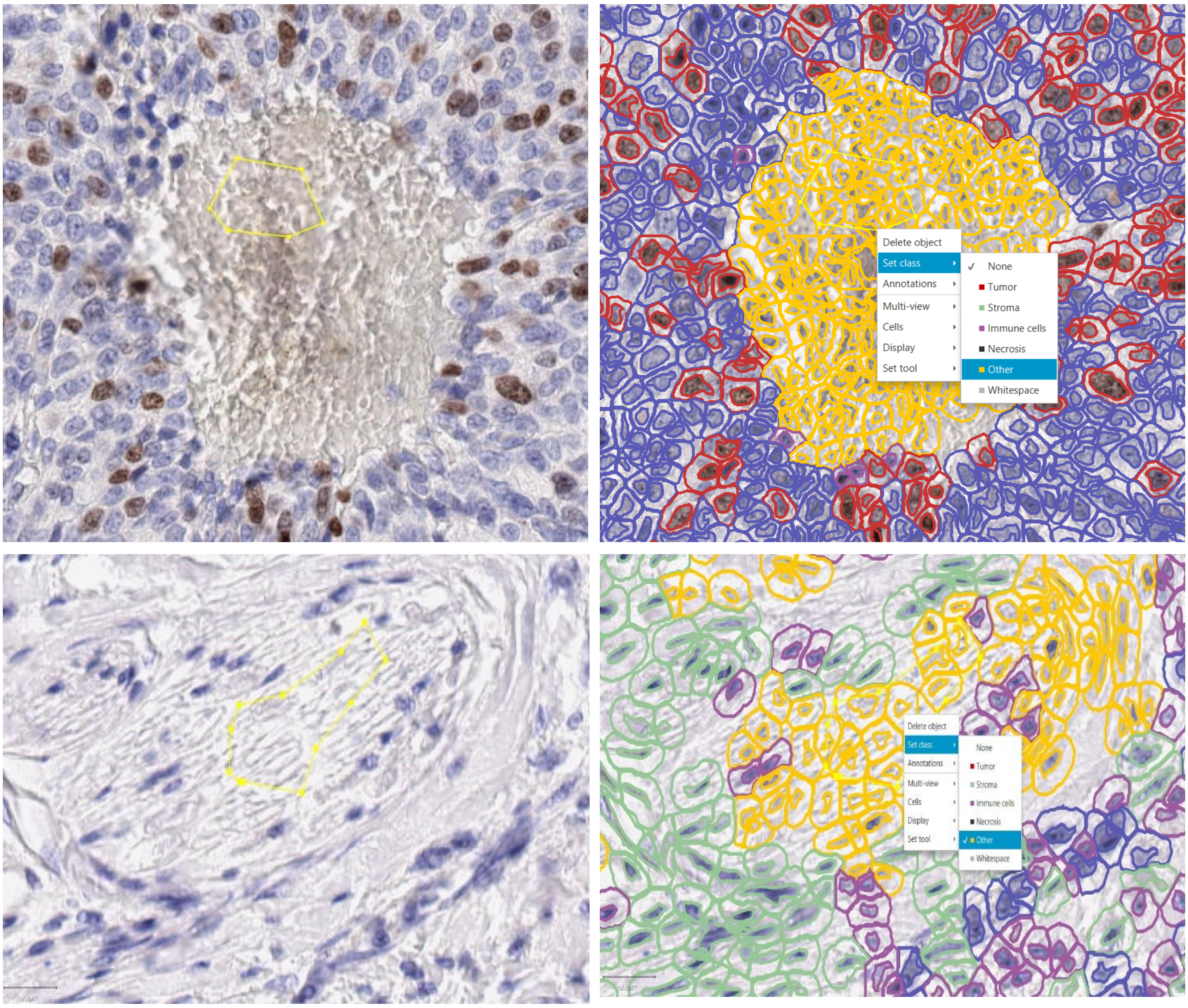

False cell detection can arise from stronger background, blood.

If you delete an annotation, make sure you keep the objects (click yes). Unless you want to exclude cells or regions from the analysis. You should exclude large number of normal epithelium, DCIS and artifacts (e.g.: irrelevant DAB staining) within invasive tumor areas that

may influence the Ki67 LI score. To do this, annotate these areas, then delete those annotations and do not keep the objects (click no). Small areas of normal tissue, DCIS do not have significant impact on Ki67 LI, when you measure in this tumor cell count range. Once you have several annotations with different classes, it is time to create the classifier to see how well QuPath can distinguish the cells. Go to *Classify/Create detection classifier*. Click on *Advanced options*, then click *Use all* button and select *Balance classes*. Pressing *Build & Apply* will train up a classifier that QuPath will then apply to all cells within the image (The color of the detected cells will correspond to color of its class).

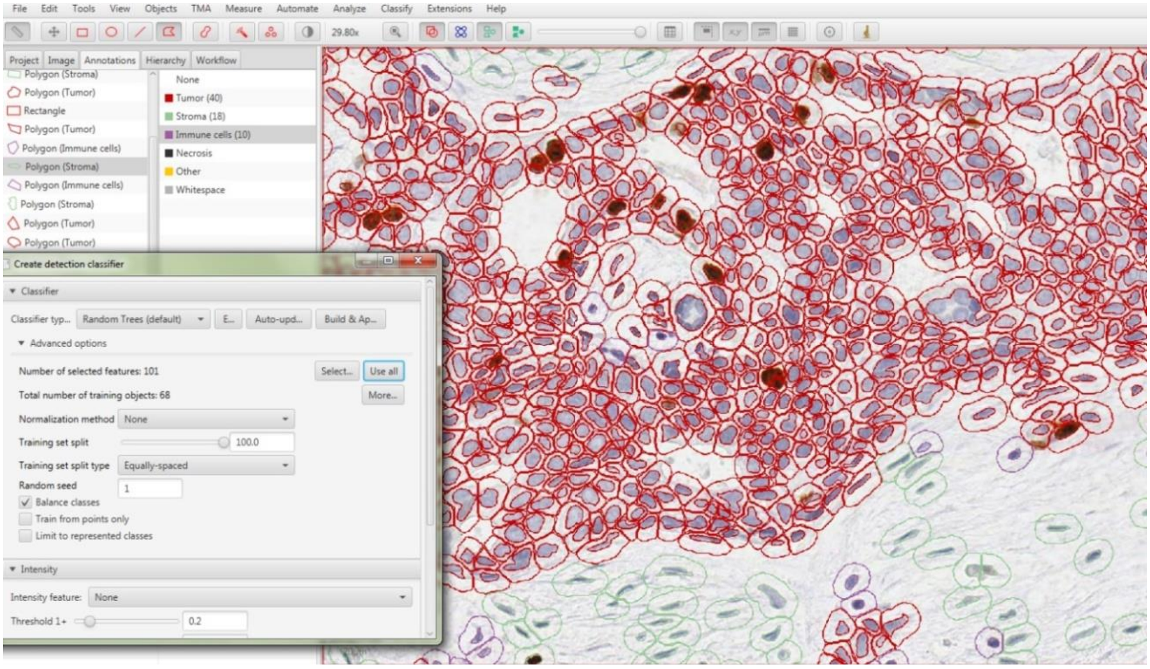

You can interactively improve the classification performance by adding annotations and set their classes in areas that QuPath gets 'wrong', until you are satisfied with the performance. After each annotation and setting its class, click *Build & Apply* to train the classifier. Carefully review the whole slide searching for major misclassifications. The color of the detected cells corresponds its class. The recommended minimum number of cell count ranges from 150 -300 annotated cells for Tumor, Stroma and Immune cells classes. Once you are satisfied with QuPath's performance to identify tumor cells, it is now time to apply DAB staining intensity classification. For the *Intensity feature*, select *Nucleus: DAB OD mean*. Set intensity threshold 1+ at 0.16 and check the box at *Use single threshold*. Then click *Build & Apply*.

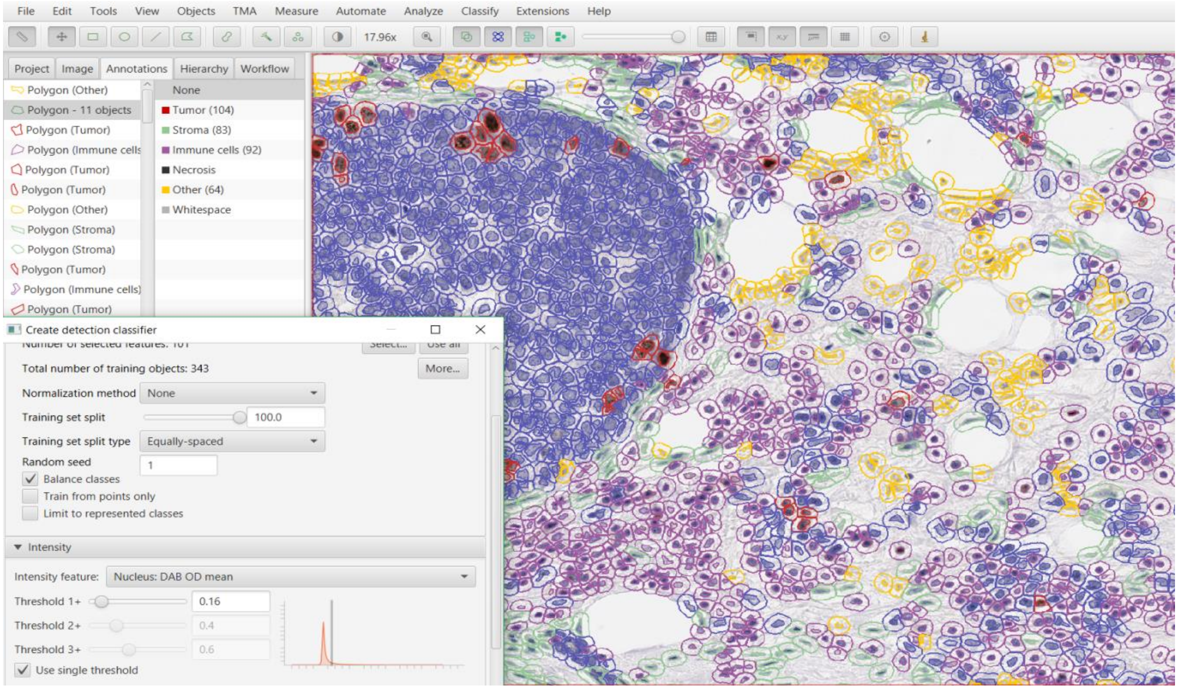

To apply the classifier again to the other slides, save this by clicking the *Save classifier* button at the bottom of the classifier window. Also save the training objects by clicking *More/Save training objects* in the classifier window. When you trained and saved the classifier and open a new image, you have the option to *Retain current training objects in classifier*, select *Yes*. When you open the next slide, you should run the estimate stain vectors, cell detection, feature calculations as before, and then apply your pre-trained classifier as follows: From the *Classify* menu select *Load classifier*. Then click *Load classifier* and select the classifier file that you saved. The classifier will be immediately applied on the opened image. The loading time depends on the image size.

Step 5: View and export results

If you select the original, large annotation containing all the cells then Ki67 labeling index will appear in the lower measurements section of the *Annotations* tab on the left of the screen as *Tumor: Positive %*. You can also generate results tables by clicking *Measure/Show annotation measurements*. Click *Copy to clipboard* and past to an excel file.

After you applied your core-biopsy trained classifier on the 30 core-biopsy slides (export results into *CB-classifer_on_CB_slides* excel sheet), please apply it on the 30 whole slides and export the results into a separate excel sheet (*CB-classifer_on_WSI* excel sheet). After you applied your whole slide trained classifier on the 30 whole slides (export results into *WSI-classifer_on_WSI* excel sheet), please apply it on the 30 core-biopsy slides and export the results into a separate excel sheet (*WSI-classifer_on_CB_slides* excel sheet). Please be noted that the classifiers are saved in the correspondent project (CB-classifier in CB project, WSI-classifier in WSI project). Finally, make sure that your excel file (see the sample excel file) contains only data of the large annotation for each slide. Name your excel file as: *YourLabName_IKBCWG_Date*. Send your results, your 2 classifiers and your 2 training objects files to Balazs Acs (balazs.acs@ki.se).

If you have any questions, please contact Balazs Acs (balazs.acs@ki.se).

Reference:

All the information regarding how to use QuPath in more details can be found on QuPath documentation website.

If you are interested in how to batch process larger numbers of images in a reproducible way, review the Automation section in QuPath documentation or contact Balazs Acs (balazs.acs@ki.se). Please be noted, that the scripts should be used with caution and attention: You should only use automation, if you understand each line in the script (groovy language).
