# Supplementary material for: Systematically higher Ki67 scores on core biopsy samples compared to corresponding resection specimen in breast cancer: a multi-operator and multi-institutional study
Source: Mod Pathol. 2022 Jun 21;35(10):1362–9. doi: 10.1038/s41379-022-01104-9 (PMC9514990; doi:10.1038/s41379-022-01104-9)
Supplement: Supplementary file 2 — Supplementary Table 1 [file 41379_2022_1104_MOESM2_ESM.pdf]

Supplementary Table 1

The list of features used in the random trees-based classification.

	Smoothed: 25 µm: Nucleus: Area	Smoothed: 50 µm: Nucleus: Area
Nucleus: Area	Smoothed: 25 µm: Nucleus: Perimeter	Smoothed: 50 µm: Nucleus: Perimeter
Nucleus: Perimeter	Smoothed: 25 µm: Nucleus: Circularity	Smoothed: 50 µm: Nucleus: Circularity
Nucleus: Circularity	Smoothed: 25 µm: Nucleus: Max caliper	Smoothed: 50 µm: Nucleus: Max caliper
Nucleus: Max caliper	Smoothed: 25 µm: Nucleus: Min caliper	Smoothed: 50 µm: Nucleus: Min caliper
Nucleus: Min caliper	Smoothed: 25 µm: Nucleus: Eccentricity	Smoothed: 50 µm: Nucleus: Eccentricity
Nucleus: Eccentricity	Smoothed: 25 µm: Nucleus: Hematoxylin OD mean	Smoothed: 50 µm: Nucleus: Hematoxylin OD mean
Nucleus: Hematoxylin OD mean	Smoothed: 25 µm: Nucleus: Hematoxylin OD sum	Smoothed: 50 µm: Nucleus: Hematoxylin OD sum
Nucleus: Hematoxylin OD sum	Smoothed: 25 µm: Nucleus: Hematoxylin OD std dev	Smoothed: 50 µm: Nucleus: Hematoxylin OD std dev
Nucleus: Hematoxylin OD std dev	Smoothed: 25 µm: Nucleus: Hematoxylin OD max	Smoothed: 50 µm: Nucleus: Hematoxylin OD max
Nucleus: Hematoxylin OD max	Smoothed: 25 µm: Nucleus: Hematoxylin OD min	Smoothed: 50 µm: Nucleus: Hematoxylin OD min
Nucleus: Hematoxylin OD min	Smoothed: 25 µm: Nucleus: Hematoxylin OD range	Smoothed: 50 µm: Nucleus: Hematoxylin OD range
Nucleus: Hematoxylin OD range	Smoothed: 25 µm: Nucleus: DAB OD mean	Smoothed: 50 µm: Nucleus: DAB OD mean
Nucleus: DAB OD mean	Smoothed: 25 µm: Nucleus: DAB OD sum	Smoothed: 50 µm: Nucleus: DAB OD sum
Nucleus: DAB OD sum	Smoothed: 25 µm: Nucleus: DAB OD std dev	Smoothed: 50 µm: Nucleus: DAB OD std dev
Nucleus: DAB OD std dev	Smoothed: 25 µm: Nucleus: DAB OD max	Smoothed: 50 µm: Nucleus: DAB OD max
Nucleus: DAB OD max	Smoothed: 25 µm: Nucleus: DAB OD min	Smoothed: 50 µm: Nucleus: DAB OD min
Nucleus: DAB OD min	Smoothed: 25 µm: Nucleus: DAB OD range	Smoothed: 50 µm: Nucleus: DAB OD range
Nucleus: DAB OD range	Smoothed: 25 µm: Cell: Area	Smoothed: 50 µm: Cell: Area
Cell: Area	Smoothed: 25 µm: Cell: Perimeter	Smoothed: 50 µm: Cell: Perimeter
Cell: Perimeter	Smoothed: 25 µm: Cell: Circularity	Smoothed: 50 µm: Cell: Circularity
Cell: Circularity	Smoothed: 25 µm: Cell: Max caliper	Smoothed: 50 µm: Cell: Max caliper
Cell: Max caliper	Smoothed: 25 µm: Cell: Min caliper	Smoothed: 50 µm: Cell: Min caliper
Cell: Min caliper	Smoothed: 25 µm: Cell: Eccentricity	Smoothed: 50 µm: Cell: Eccentricity
Cell: Eccentricity	Smoothed: 25 µm: Cell: DAB OD mean	Smoothed: 50 µm: Cell: DAB OD mean
Cell: DAB OD mean	Smoothed: 25 µm: Cell: DAB OD std dev	Smoothed: 50 µm: Cell: DAB OD std dev
Cell: DAB OD std dev	Smoothed: 25 µm: Cell: DAB OD max	Smoothed: 50 µm: Cell: DAB OD max
Cell: DAB OD max	Smoothed: 25 µm: Cell: DAB OD min	Smoothed: 50 µm: Cell: DAB OD min
Cell: DAB OD min	Smoothed: 25 µm: Cytoplasm: DAB OD mean	Smoothed: 50 µm: Cytoplasm: DAB OD mean
Cytoplasm: DAB OD mean	Smoothed: 25 µm: Cytoplasm: DAB OD std dev	Smoothed: 50 µm: Cytoplasm: DAB OD std dev
Cytoplasm: DAB OD std dev	Smoothed: 25 µm: Cytoplasm: DAB OD max	Smoothed: 50 µm: Cytoplasm: DAB OD max
Cytoplasm: DAB OD max	Smoothed: 25 µm: Cytoplasm: DAB OD min	Smoothed: 50 µm: Cytoplasm: DAB OD min
Cytoplasm: DAB OD min	Smoothed: 25 µm: Nucleus/Cell area ratio	Smoothed: 50 µm: Nucleus/Cell area ratio
Nucleus/Cell area ratio	Smoothed: 25 µm: Nearby detection counts	Smoothed: 50 µm: Nearby detection counts
